# Which Head and Neck Cancer Patients Are Most at Risk of High Levels of Fear of Cancer Recurrence

**DOI:** 10.3389/fpsyg.2021.671366

**Published:** 2021-07-16

**Authors:** Simon N. Rogers, Camilla Monssen, Gerald M. Humphris, Derek Lowe, Anastasios Kanatas

**Affiliations:** ^1^Faculty of Health and Social Care, Edge Hill University, Ormskirk, United Kingdom; ^2^Liverpool Head and Neck Centre, Liverpool University Hospital Aintree, Liverpool, United Kingdom; ^3^School of Medicine, Medical and Biological Sciences, North Haugh, St. Andrews, United Kingdom; ^4^Astraglobe Ltd., Congleton, United Kingdom; ^5^Leeds Teaching Hospitals and St. James Institute of Oncology, Leeds Dental Institute and Leeds General Infirmary, Leeds, United Kingdom

**Keywords:** fear of cancer recurrence, quality of life, patient concerns inventory, head and neck cancer, randomized trial

## Abstract

**Background:** Fear of cancer recurrence (FCR) is recognized as a common concern for patients with head and neck cancer (HNC). The aim of this study is to describe in greater detail the demographic and clinical characteristics of HCN patients who indicate a high level of FCR in their review consultation.

**Methods:** A pragmatic cluster-controlled trial was conducted between January 2017 and December 2018 at two UK HNC centers (Leeds and Liverpool) to test the efficacy of a prompt tool called the Patient Concerns Inventory (PCI). Patients completed the PCI and the UW-QOLv4 which included a single 5 category rating of FCR. Secondary statistical analyses focused on variables associated with high FCR.

**Results:** Two hundred and eighty-eight trial patients were recruited in this trial. At a median of 194 days after diagnosis and 103 days after the end of treatment 8% stated (*n* = 24) “I get a lot of fears of recurrence and these can really preoccupy my thoughts” and 3% (*n* = 8) “I am fearful all the time that my cancer might return, and I struggle with this.” Thus, 11% (*n* = 32) responded in the worst two categories, 95% Confidence interval 7.7–15.3% for high FCR. Stepwise logistic regression resulted in female gender (*p* < 0.001), age (*p* = 0.007), and receiving financial benefits (*p* = 0.01) as independent predictors.

**Conclusions:** Around one in ten HNC patients attending routine outpatient follow-up consultations report high FCR, however for female patients under the age of 55 the rate was one in three. This group requires specialist attention and could be the focus of a multicenter intervention trial.

## Introduction

Head and neck cancer (HNC) is the eighth most commonly diagnosed cancer in the UK (Cancer Research UK, 2017) with approximately 12,200 new cases each year. Risk factors include smoking and alcohol and recently the Human Papilloma virus (HPV) (Cooper et al., 2009; Dhull et al., 2018). The 5-year disease specific survival is around 60% for both men and women (Cadoni et al., 2017). Most HNC recurrences occur within the first 2 years following diagnosis (Kissun et al., 2006) and have a poor prognosis with survival in terms of months (Fullarton et al., 2016). With this backdrop it is hardly surprising that Fear of Cancer Recurrence (FCR) is (Humphris and Ozakinci, 2008) one of the most frequent issues patients wish to talk about in their out-patient review consultations (Kanatas et al., 2012). Given the likelihood of early recurrence, clinicians often stress the importance of vigilance and adherence to follow-up visits. One of the greatest challenges is carefully balancing the degree of emphasis on recurrence without alarming the patient. Fear of Cancer Recurrence is of particular interest as this fear holds considerable psychological stress which in turn negatively affects the patient's quality of life (QoL) (Smith et al., 2006; Dunne et al., 2017), daily functioning (Lee-Jones et al., 1997), relationship with carers (Hodges and Humphris, 2009), and mental well-being (Humphris et al., 2003). HNC survivors with inadequate health literacy have increased FCR compared to those with adequate health literacy (Clarke et al., 2021).

The issue of FCR can be hard to elicit in the follow-up clinic, whether this be face to face or virtual, as imposed by restrictions due to the COVID-19 pandemic. The Patients Concerns Inventory (PCI) is a condition specific prompt list devised in collaboration with patients as a means by which they can raise issues of concern with the clinician (Rogers et al., 2009) (Supplementary Material). A systematic review and content comparison of unmet needs self-report measures favored the PCI over 13 other tools (Shunmugasundaram et al., 2019). In a randomized trial the PCI has been shown to be a low-cost intervention which is feasible in routine clinical practice and is associated with a positive effect on QoL and socio-emotional dysfunction (Rogers et al., 2020).

Although the PCI can help to identify the frequency of FCR (Rogers et al., 2010a; Kanatas et al., 2012; Ghazali et al., 2013), thus far specific details of which kind of patient is most at risk are lacking. The PCI randomized trial (Rogers et al., 2020) has given an opportunity to evaluate in much greater depth the issue of FCR and this is of particular merit as the trial is based in standard practice and involved 15 consultant HNC surgeons. Hence, the aim of this study is to describe the demographic background and clinical characteristics for those HCN patients who indicate a high level of FCR in their review consultation. This may allow prompt identification of those patients in need of further support during their clinical consultations.

## Materials and Methods

### Setting and Participants

The methodology of this trial has been described in detail (Rogers et al., 2018a). Briefly, this is a pragmatic cluster-controlled trial, with consultants (clusters) randomized to “using” or “not using” an intervention incorporating the PCI prompt list at all their trial clinics, i.e., at both baseline and at all follow-up clinics. Two centers participated, Aintree and Leeds, with 15 consultants of whom 8 used the PCI and 7 did not. We report results from the first “baseline” trial clinic and focus on findings regarding fear of recurrence (FCR). We also report FCR results after 12 months of follow-up. Eligible patients were treated curatively for primary HNC, and all sites, stages of disease and treatments were included, and second primary tumors were later accepted. Patients treated palliatively or with a recurrence or with a history of cognitive impairment, psychoses or dementia were excluded.

### Measures

The UW-QOL v4 questionnaire comprises 12 single item domains, with between 3 and 5 response options scaled evenly from 0 (worst) to 100 (best) according to response hierarchy (Rogers et al., 2002). UW-QOL domains are presented within two subscales, physical function and social-emotional function (Rogers et al., 2010b) with each subscale score being the mean of six domain scores (http://www.hancsupport.com/professionals/quality-life/qol-questionnaires/university-washington). The physical function score is the mean of the appearance, swallowing, chewing, speech, taste and saliva domain scores, while the social-emotional score is the mean of the pain, activity, recreation, shoulder, mood, and anxiety domain scores. Criteria derived from earlier work was used to highlight domains in which patients have a significant problem or dysfunction (Rogers and Lowe, 2009). There was also a single item overall QOL question on the UWQOL v4 for which patients are asked to consider not only physical and mental health, but also other factors, such as family, friends, spirituality, or personal leisure activities important to their enjoyment of life. The study HRQOL data also included the Distress Thermometer score (Hegel et al., 2008) and EQ-5D-5L (Rogers et al., 2016).

The FCR question has five response options: (A) I have no fear of recurrence, (B) I have a little fear, with occasional thoughts but they don't really bother me, (C) I am sometimes having fearful thoughts, but I can usually manage these, (D) I get a lot of fears of recurrence and these can really preoccupy my thoughts, (E) I am fearful all the time that my cancer might return and I struggle with this. There is also a separate question asking patients whether FCR had been important to them (Yes/No) during the past 7 days. From earlier work (Rogers et al., 2016) it was postulated that those patients responding in the worst two categories should be considered for added assessment and support; we define these patients as having a significant problem or dysfunction with FCR.

The PCI is a condition-specific item prompt list (Rogers et al., 2009) comprising 56 items, which patients select from before seeing their consultant, to help guide the outpatient consultation, and it covers a range of symptoms and potential problems patients may face after treatment. It helps focus the consultation, aids doctor–patient communication, and helps route patients to other professionals for advice and support. It can be integrated into routine clinical practice (Rogers et al., 2018b).

Pre-consultation questionnaires including the PCI prompt list were completed electronically (desktop, tablet, iPAD) apart from one Liverpool hospital (non-PCI consultant) that used paper. PCI patients took into their consultations a summary sheet of paper that listed (a) all PCI items they selected for discussion, (b) any University of Washington (UWQOL) questionnaire domains in which there was a significant problem or dysfunction, (c) their overall QOL response, (d) their Distress Thermometer score, and (e) health professionals they wanted to see. This one page paper summary printout was the visible difference between trial arms as far as contact between consultant and patient was concerned. Control patients completed exactly the same pre-clinic information apart from the PCI prompt list but neither they nor their consultant saw any summary sheet. Both groups completed the EQ-5D-5L for purposes of health economic assessment.

Baseline clinical and demographic data were collected using a baseline clinic questionnaire based on that of the Head and Neck 5000 project (Ness et al., 2014), or by extraction from baseline clinical records. Information was collected as to whether patients lived alone or with others, whether they were working and whether they lived in a household that received UK state financial benefits. Lifestyle factors about tobacco and alcohol use were also collected, as was patient gender and age. Clinical details about primary tumor site, grade, treatment, WHO, and ACE27 comorbidity were obtained from clinical records. Index of Multiple Deprivation (English Indices of Deprivation–IMD, 2019) scores were derived from patient postcodes using publicly available data and these provide a relative measure of deprivation at a small area level across England.

### Statistical Analyses

The statistical analysis focused on variables associated with FCR dysfunction. We considered patient and clinical casemix variables and also a wide range of HRQOL measures. Fishers Exact test was used to compare patient groups regarding FCR dysfunction (Yes/No). Logistic regression was used to form predictive models of FCR dysfunction. Univariate models with each predictor variable were run and the Nagelkerke R square value (*R*^2^) was noted for each model, these values providing an indication of which models better predict FCR dysfunction; the higher the value the better the prediction. Multivariable logistic regression modeling was done using significant univariate casemix variables (Table 1) and a stepwise regression approach with *p* < 0.01 for inclusion was adopted for the consideration of the many HRQOL predictor variables (Table 2). Given the number of tests performed for this paper, statistical significance was regarded as *p* < 0.01.

**Table 1:** Patient and clinical characteristics and fear of recurrence.

			Fear of recurrence response category^*^					**% with FCR dysfunction (A&B)**	*P*-value^***^	*R*^**2**^-value^****^
		**Patients**	**A&B)**	**C+**	**C–**	**D**	**E**			
	Total	288	32	43	43	127	43	11		
Trial group	PCI	140	16	19	24	62	19	11	>0.99	<0.1
	Non-PCI	148	16	24	19	65	24	11		
Hospital	Aintree	178	19	30	26	76	27	11	0.85	0.1
	Leeds	110	13	13	17	51	16	12		
Days from diagnosis to first clinic (TERTILES)	≤144	96	12	14	14	39	17	13	0.60	
	125–227	96	8	15	18	41	14	8		0.8
	≥228	96	12	14	11	47	12	13		
Days from end of treatment to first clinic (TERTILES)	≤79	96	13	13	16	37	17	14	0.29	1.8
	80–138	98	7	16	15	47	13	7		
	≥139	94	12	14	12	43	13	13		
Duration of consultation (min) TERTILES	≤8 min	92	7	9	13	38	25	8		1.8
	9–12 min	103	10	17	16	47	13	10	0.32	
	≥13 min	88	13	17	14	39	5	15		
Gender	Female	90	19	18	16	26	11	21	0.001	8.3
	Male	198	13	25	27	101	32	7		
Age	<55	71	15	12	13	27	4	21	0.002	10.3 (<55, 55–64, ≥65)
	55–64	116	14	19	18	50	15	12		
	65–74	67	3	7	10	35	12	4		
	≥75	34	0	5	2	15	12	0		
Tumor site:	Oral cavity	134	18	18	23	57	18	13	0.77	1.0
	Oropharynx	91	8	20	12	37	14	9		
	Larynx	41	4	3	4	21	9	10		
	Other	22	2	2	4	12	2	9		
Overall clinical stage	Advanced 3–4	164	18	29	26	71	20	11	>0.99	<0.1
	Early 1–2	124	14	14	17	56	23	11		
Primary treatment^**^:	S only	95	12	9	10	44	20	13	0.93	
	S only and FF	21	1	5	4	8	3	5		
	RT or RT/CT only	58	7	11	11	24	5	12		1.0
	S and (RT or RT/CT)	68	7	13	9	31	8	10		
	S and (RT or RT/CT) and FF	46	5	5	9	20	7	11		
WHO comorbidity	0	179	15	31	27	82	24	8	0.10	
	1	67	9	6	11	32	9	13		2.8
	2–4	42	8	6	5	13	10	19		
ACE27 comorbidity	None	137	15	23	20	59	20	11	0.50	1.1
	Mild	95	11	14	13	43	14	12		
	Moderate	48	4	6	8	22	8	8		
	Severe	8	2	0	2	3	1	25		
Living situation	Alone in house/flat	65	4	8	7	29	17	6	0.26	
	With others in house/flat	220	27	35	36	97	25	12		1.5
Working	Yes	88	7	15	9	46	11	8	0.31	0.9
	No	192	24	27	31	79	31	13		
Financial (household) benefits	Yes	107	17	14	18	44	14	16	0.01	4.8
	No	158	10	26	20	77	25	6		
Smoking habit	Current	37	9	2	11	10	5	24	0.009	
	Former	163	19	27	22	72	23	12		6.1
	Never	80	4	13	9	41	13	5		
Alcohol habit	Current	194	20	26	29	89	30	10	0.65	
	Former	73	9	13	11	31	9	12		0.3
	Never	13	2	3	2	3	3	15		
IMD 2019 quintile	1 = least deprived	34	3	7	5	14	5	9	0.73	
	2	55	4	13	7	20	11	7		
	3	49	5	7	8	23	6	10		1.7
	4	40	4	4	4	24	4	10		
	5 = most deprived	110	16	12	19	46	17	15		

* *(A), I have no fear of recurrence; (B), I have a little fear, with occasional thoughts but they don't really bother me; (C+), I am sometimes having fearful thoughts but I can usually manage these, and important during the past 7 days; (C–), I am sometimes having fearful thoughts but I can usually manage these and NOT important during the past 7 days; (D), I get a lot of fears of recurrence and these can really preoccupy my thoughts; (E), I am fearful all the time that my cancer might return and I struggle with this*.

** *Surgery (S), RadioTherapy (RT), ChemoTherapy (CT), Free Flap transfer (FF)*.

*** *Fishers Exact test*.

**** *Nagelkerke R^2^-value provides an indication of which models better predict FCR dysfunction; the higher the value the better the prediction*.

**Table 2:** HRQOL measures and fear of recurrence.

			Fear of recurrence response category^*^					**% with FCR dysfunction (A&B)**	*P*-value^***^	*R*^**2**^-value^****^
		**Patients**	**A&B)**	**C+**	**C–**	**D**	**E**			
	Total	288	32	43	43	127	43	11		
UWQOL Overall Quality of life	Outstanding/Very good	105	4	9	12	58	22	4	<0.001	16.1
	Good	94	9	20	12	41	12	10		
	Fair	63	8	11	15	23	6	13		
	Very Poor/Poor	26	11	3	4	5	3	42		
Distress thermometer (DT)	Zero	79	0	3	4	46	26	0	<0.001	17.5 (0–3, 4–5, 6–10)
	1–3	80	5	14	13	39	9	6		
	4–5	60	9	12	7	27	5	15		
	6–10	69	18	14	19	15	3	26		
UWQOL social-emotional subscale	<60	72	24	14	16	17	1	33	<0.001	26.7
	60–79	98	5	18	18	46	11	5		
	80–100	118	3	11	9	64	31	3		
UWQOL physical function subscale	<60	91	20	18	18	27	8	22	0.001	
	60–79	108	8	14	17	53	16	7		10.4
	80–100	89	4	11	8	47	19	4		
**Social-emotional subscale**										
∙ Pain	Best possible response	108	8	12	7	56	25	7	0.20	
	Somewhere in-between	97	11	14	22	37	13	11		2.2
	Dysfunction	83	13	17	14	34	5	16		
∙ Activity	Best possible response	86	3	7	9	46	21	3	<0.001	
	Somewhere in-between	168	19	31	25	74	19	11		10.2
	Dysfunction	34	10	5	9	7	3	29		
∙ Recreation	Best possible response	109	8	9	10	58	24	7	<0.001	
	Somewhere in-between	156	14	28	32	64	18	9		12.1
	Dysfunction	23	10	6	1	5	1	43		
∙ Shoulder	Best possible response	160	6	25	26	73	30	4	<0.001	
	Somewhere in-between	92	18	14	13	38	9	20		13.8
	Dysfunction	36	8	4	4	16	4	22		
∙ Mood	Best possible response	97	0	4	6	58	29	0	<0.001	
	Somewhere in-between	146	11	34	26	63	12	8		31.2 (dysfunction, other)
	Dysfunction	45	21	5	11	6	2	47		
∙ Anxiety	Best possible response	103	1	2	4	56	40	1	<0.001	
	Somewhere in-between	136	7	28	31	68	2	5		41.1
	Dysfunction	49	24	13	8	3	1	49		
**Physical function subscale**										
∙ Appearance	Best possible response	75	3	10	9	30	23	4	0.003	
	Somewhere in-between	185	21	28	28	91	17	11		7.7
	Dysfunction	28	8	5	6	6	3	29		
∙ Swallowing	Best possible response	104	9	12	10	54	19	9	0.03	
	Somewhere in-between	142	13	23	22	64	20	9		4.5
	Dysfunction	42	10	8	11	9	4	24		
∙ Chewing	Best possible response	117	8	11	13	63	22	7	0.09	
	Somewhere in-between	133	17	28	18	54	16	13		3.1
	Dysfunction	38	7	4	12	10	5	18		
∙ Speech	Best possible response	123	11	15	13	60	24	9	0.02	
	Somewhere in-between	142	14	25	24	62	17	10		4.9
	Dysfunction	23	7	3	6	5	2	30		
∙ Taste	Best possible response	90	3	9	11	50	17	3	<0.001	
	Somewhere in-between	141	20	23	21	59	18	14		6.6
	Dysfunction	57	9	11	11	18	8	16		
∙ Saliva	Best possible response	80	3	6	7	43	21	4	0.03	
	Somewhere in-between	109	14	21	17	42	15	13		5.1
	Dysfunction	99	15	16	19	42	7	15		
**Other item:**										
∙ Intimacy	Best possible response	212	13	25	26	108	40	6	<0.001	
	Somewhere in-between	61	15	15	12	16	3	25		11.9
	Dysfunction	15	4	3	5	3	0	27		
**EQ-5D**										
Mobility (walking about)	No problems	180	12	22	23	93	30	7	0.002	
	Slight problems	43	5	9	6	14	9	12		7.9
	Moderate/severe/unable	65	15	12	14	20	4	23		
Self-care (washing or dressing myself)	No problems	222	15	34	30	103	40	7	<0.001	
	Slight problems	34	5	4	5	17	3	15		13.6
	Moderate/severe/unable	32	12	5	8	7	0	38		
Usual activities	No problems	143	9	16	16	73	29	6	0.001	
	Slight problems	74	6	14	13	33	8	8		9.4
	Moderate/severe/unable	71	17	13	14	21	6	24		
Pain (or discomfort)	No pain or discomfort	104	7	10	8	52	27	7	0.04	
	Slight pain or discomfort	96	9	17	17	43	10	9		4.4
	Moderate/severe/extreme	88	16	16	18	32	6	18		
Anxiety/Depression	Not anxious or depressed	145	1	4	19	83	38	1	<0.001	
	Slightly anxious or depressed	95	10	30	14	39	2	11		37.0
	Moderate/severe/extreme	48	21	9	10	5	3	44		
EQ-5D-5L TTO crosswalk values (TERTILES)	≤0.6950	96	24	19	18	32	3	25	<0.001	
	0.6951–0.8370	109	3	21	18	48	19	3		18.3
	≥0.8371	83	5	3	7	47	21	6		
EQ5D Visual analog scale (VAS) TERTILES	≤69	96	19	15	20	36	6	20	0.007	
	70–81	96	7	23	11	37	18	7		7.1
	≥82	96	6	5	12	54	19	6		

* *(A), I have no fear of recurrence; (B), I have a little fear, with occasional thoughts but they don't really bother me, (C+), I am sometimes having fearful thoughts but I can usually manage these, and important during the past 7 days, (C–), I am sometimes having fearful thoughts but I can usually manage these, and NOT important during the past 7 days, (D), I get a lot of fears of recurrence and these can really preoccupy my thoughts, (E), I am fearful all the time that my cancer might return and I struggle with this*.

*** *Fishers Exact test*.

**** *Nagelkerke R^2^-value provides an indication of which models better predict FCR dysfunction; the higher the value the better the prediction*.

## Results

Patients for the trial were first discussed at MDT meetings between January 2017 and December 2018, with first trial clinics between April 2017 and October 2019. Characteristics of the 288 trial patients can be determined from Table 1. Median (IQR) age at baseline was 62 (55–69) and 69% (198) were male. Baseline clinics were a median (IQR) of 194 (125–249) days after diagnosis and 103 (71–162) days after the end of treatment. Regarding FCR, 15% (43) stated “I have no fear of recurrence,” 44% (127) “I have a little fear, with occasional thoughts but they don't really bother me,” 30% (86) “I am sometimes having fearful thoughts but I can usually manage these,” 8% (24) “I get a lot of fears of recurrence and these can really preoccupy my thoughts,” and 3% (8) “I am fearful all the time that my cancer might return and I struggle with this.” Thus 11% (32) responded in the worst two categories, 95% Confidence interval 7.7–15.3% for FCR dysfunction, and this rate was notably higher in females and in patients younger than 65 years (Table 1). No significant variation was seen regarding tumor site, clinical stage, or treatment. It was also higher in those still smoking and in those with households receiving financial benefits. Stepwise logistic regression with gender, age (<55/55–64/≥65), financial benefits (Yes/No), and smoking habit (Current/Ex/Never) resulted in gender (*p* < 0.001), age (*p* = 0.007), and financial benefits (*p* = 0.01) as independent predictors, *n* = 265. The first two predictors, gender and age, were retained to preserve the full sample size. In female patients under the age of 55 the rate was 36% (10/28), while for those aged 55–64 it was 29% (8/28) and for those ≥65 it was 3% (1/34); for males the rates were 12% (5/43), 7% (6/88), and 3% (2/67), respectively.

Most of the HRQOL measures in this study were associated with FCR (Table 2) and with each other (results not shown). Fear of Cancer Recurrence dysfunction was present in 49% (24/49) of those with dysfunction in anxiety (UWQOL), in 47% (21/45) of those with dysfunction in mood (UWQOL), in 44% (21/48) with moderate, severe, or extreme problems with anxiety/depression (EQ5D), in 43% (10/23) with dysfunction in recreation (UWQOL) and 42% (11/26) with poor or very poor overall quality of file (UWQOL). Comparison of the *R*^2^ values indicated that the most predictive variables from Table 2 were UWQOL anxiety (*R*^2^ = 41.1), EQ5D anxiety/depression (37.0), UWQOL Mood (31.2) and UWQOL social-emotional function subscale score (26.9). In comparison the *R*^2^-value for the model with gender and age group was 18.2. When gender and age and all the variables from Table 2 were considered within a stepwise regression at *p* < 0.001 for entry then UWQOL anxiety (*p* < 0.001, *R*^2^ = 41.1) was selected first, followed by UWQOL Mood (*p* < 0.001, *R*^2^ = 46.4 combined) and then gender (*p* = 0.002, *R*^2^ = 52.1 combined). If patients had both UWQOL anxiety and mood dysfunction then 66% (19/29) also had FCR dysfunction (males: 61%, 11/18; females: 73%, 8/11). If patients had UWQOL anxiety and mood dysfunction but not both then 19% (7/36) had FCR dysfunction (males; 9%, 2/22; females: 36%, 5/14). For other patients only 3% (6/223) had FCR dysfunction (males: 0%, 0/158; females: 9%, 6/65).

One-third (97/288) of patients said that FCR had been important to them in the past week, and this varied from 81% (26/32) of those with FCR dysfunction, 50% (43/86) of those sometimes having fearful thoughts, 19% (24/127) of those having a little fear and 9% (4/43) of those without fear at the time of data entry. In the group who sometimes had fearful thoughts, there were no significant differences in patient characteristics between the 43 patients regarding FCR as important and the 43 patients for whom it was not important (Tables 1, 2) apart from an association with EQ5D anxiety/depression (*p* < 0.001) in Table 2 which only affected the balance between not being anxious or depressed and being slightly anxious or depressed. Patients in the PCI intervention group could select the FCR item from the PCI prompt list if they wanted to discuss this during their consultation, and 34% (48/140) did select this item. Of the 16 patients with FCR dysfunction then 8 wanted to discuss their fears and 8 did not (Table 3). About half (53%, 23/43) of patients who sometimes had fearful thoughts but could usually manage them wanted to discuss their fears, 74% (14/19) if FCR had been important to them during the past week and 38% (9/24) if FCR had not been not important. About one quarter (27%, 17/62) with just a little fear wanted to discuss FCR, 56% (5/9) if FCR had been important, and 23% (12/53) if not important. None of the 19 patients without FCR wanted to discuss the issue. Of those wanting to discuss their fears, most (40/48) did not have FCR dysfunction.

**Table 3:** Selection of FCR item from the PCI prompt list for 140 PCI patients, by self-reported level of fear of recurrence (FCR) and by whether FCR had been important over the past 7 days.

**Single FCR question**	**FCR item selected from PCI prompt list**		**FCR important in the past 7 days**	**FCR item selected from PCI prompt list**	
	**%**	**Patients**		**%**	**Patients**
I am fearful all the time that my cancer might return and I struggle with this	50	8/16	Yes	57	8/14
I get a lot of fears of recurrence and these can really preoccupy my thoughts			No	0	0/2
I am sometimes having fearful thoughts but I can usually manage these	53	23/43	Yes	74	14/19
			No	38	9/24
I have a little fear, with occasional thoughts but they don't really bother me	27	17/62	Yes	56	5/9
			No	23	12/53
I have no fear of recurrence	0	0/19	Yes	0	0/1
			No	0	0/18
Total	34	48/140	Yes	63	27/43
			No	22	21/97

During the trial patients attended a median (IQR) of 4 (3–5) clinics, range 1–10. Follow-up results at around 12 months were available for 205 patients, at a median (IQR) of 357 (329–391) days from baseline. The percentages with FCR dysfunction were 8% (17/205) at the first study clinic and 6% (13/205) after about 12 months. Five patients had FCR dysfunction on both occasions, 12 only at first, 8 only later, and 180 on neither occasion. In female patients under the age of 65 the rates for FCR dysfunction were 25% (10/40) and 13% (5/40), while for older females they were 0% (0/22) and 5% (1/22); for males 6% (6/96), 7% (7/96), and 2% (1/47), 0% (0/47), respectively. Of 83 lost to the study between baseline and follow-up, 18% (15) had significant fears at the first clinic. The FCR item from the PCI prompt list was selected by 36% (36/100) at baseline and 17% (17/100) at follow-up.

## Discussion

Fear of Cancer Recurrence is an issue that is common in HNC and one that is amenable to intervention (Humphris and Rogers, 2012). Its prevalence rate overall was in the region of one in ten patients and this is similar to previous studies (Llewellyn et al., 2008). A more recent survey in the Netherlands using the Cancer Worry Scale has reported “approximately one in two of all patients newly diagnosed with HNC had FCR levels above the validated cut-off for high FCR shortly after diagnosis (Mirosevic et al., 2019).” There may be differences with the proportion of patients categorized as stating they have high FCR according to the measure employed. For example, the Mirosevic et al. article (Mirosevic et al., 2019) set their cut-off against the validated Fears of Cancer Recurrence Inventory (FCRI). A review of the FCRI has raised concerns about the low cut-off on this measure (Smith et al., 2020). Hence, careful attention needs to be paid to the actual wording and psychometric properties of the measures used. Our single item follows the principles of keeping the assessment of FCR focused and with face valid wording, expressed by Costa (2017). Furthermore, our group subscribe to the view that moderate levels of FCR are probably of value, and not regarded as dysfunctional, as the patient remains reasonably vigilant to changes to symptom experience. The caveat would be for patients to have the support from the H&N team should these fears become heightened, and maintained, and for the staff to monitor and discuss their FCR to reverse back to a moderate level. Among the variables that clinicians can use to identify patients with H&N cancer and high FCR indicated by this data set, include the following:

### Age

A significant relationship between the younger age of these patients and dysfunctional FCR was found. This was 21% in those under 55, 14% in those aged 55–64 and only 3% in those over 65. The median age in this study was 62 implying that nearly half of the patient population is at higher risk. This association is in line with previous research indicating age was a predictor of FCR (Humphris et al., 2003; Lim and Humphris, 2020). There are several potential explanations as to why age plays a significant role in fears of recurrence. Researchers (Hutton and Williams, 2001) have argued that older patients are not always able to differentiate the interference caused by treatment and the natural degeneration of aging, with a cancer diagnosis becoming increasingly more predictable with older age. In comparison, younger patients find the diagnoses of cancer much more unanticipated and abrupt as it threatens their ability to fulfill major life events, such as having grandchildren or marriage of children (Simard et al., 2013).

### Gender

The finding that gender is associated with FCR has been highlighted in a recent systematic review (Pang and Humphris, 2021). One explanation as to why females experience higher FCR is because they tend to experience more symptoms of dysfunctional anxiety (Faravelli et al., 2013). A Norwegian and Swedish HNC study found that those who were under 65 and female were the most likely to develop anxiety (Hammerlid et al., 1999). In this analysis' parent study, 17% of patients experienced dysfunctional anxiety symptoms according to the UWQOL social-emotional subscale. As anxiety levels were not included in their secondary analysis, we must leave room to consider whether present FCR are a surface level manifestation of other psychiatric comorbidities that have been previously found to be significant within this sub-population.

### Financial Benefits

In this study, it was found that a significant predictor of developing FCR was living in a household in receipt of financial benefits. Head and neck cancer has a serious impact on patient finances and is associated with poor HRQOL (Rogers et al., 2012). Previous evidence has shown that rates of financial instability are the highest within HNC in comparison to all other cancer types (Avis et al., 2005). This is exemplified within this study with 37% of participants in households receiving financial benefits. A systematic review of FCR found that of the 10 studies investigating financial difficulties and FCR, the association was only found to be significant in three (Simard et al., 2013). However, previous studies regarding FCR and financial position should be applied carefully as this fear could be more prominent in countries whose national health programme relies solely on private hospitals. In a previous study within our group, it was found that FCR had a weak relationship with deprivation which would partially support this finding of patients reporting to receive state benefits (Rylands et al., 2016). This mirrors the findings of a study by Clarke et al. (2021) in which HNC survivors with inadequate health literacy reported lower levels of self-management behaviors, lower functional HRQOL, and increased FCR.

### Health Related Quality of Life

Functional/symptom items on H&N cancer HRQOL questionnaires have potential utility in respect to FCR as symptoms that patients experience might trigger anxiety regarding the possibility of recurrence. This is especially so in the field of H&N cancer care where previous reports of patient experience of physical symptoms have focused our group to build on the FCR model proposed by Lee-Jones et al. (1997) and promoted the AFTER intervention (Humphris and Ozakinci, 2008) to reduce high FCR. Most of the HRQOL measures in this study were associated with FCR, for example, 42% in those with poor or very poor overall QOL had dysfunctional FCR. There was anxiety or mood dysfunction in 65 patients (23% of the whole sample) and this group accounted for 81% (26/32) of all those with FCR dysfunction. These relatively broad-brush HRQOL questionnaires, such as the UW-QOL mood and anxiety items and, interestingly a similar amount of variance explained with another well-developed measure, namely: the EQ-5D, together with the EORTCc30, these assessments have shown the ability to identify patients with high FCR. Due to the high degree of overlap between these HRQOL measures, only one or, at most, two would be required to assist with identifying patients with possible high FCR. Within England the NHS England QOL Metric study outcome measures include the EQ-5D and EORTCc30 (NHS England Cancer Data, 2021). Hence, it would enable all cancer patients in England to be given the opportunity to complete the questionnaires at the 18 month post diagnosis window. Those scoring badly, for example, on the EQ-5D will have possibly high FCR which would need to be further assessed. These assessment approaches are part of a possible development to construct stepped-care programmes of support and intervention for moderate to high scorers of FCR which is being explored in patients with melanoma (Lynch et al., 2020).

The regression *R*^2^-values (Table 2) indicated that the age and gender associations with FCR were dwarfed relatively by the associations between the HRQOL measures and FCR. Logistic regression on FCR using all variables in Table 2 as predictors selected anxiety, mood, and gender. For patients with anxiety or mood dysfunction or both—there was dysfunctional FCR for 56% of females (13/25) and 32% (13/40) of males; for patients without anxiety of mood dysfunction—there was dysfunctional FCR for 9% (6/65) of females and 0% (0/158) of males.

Our results have included a split of the FCR patient responses to the middle category of the rating scale. The split was whether, or not, FCR had been “important” to the patient within the previous 7 days. We found on close inspection little difference about this split—in either patient/clinical characteristics or across the HRQOL measures. Hence the important message that this reinforces is that dysfunction in FCR is best measured by the worst two FCR response options only and that the middle option if important should not be added to this. This recommendation was postulated in a previous paper (Rogers et al., 2016). In addition, it is worth emphasizing that a degree of FCR is “normal” and to be expected. In our sample, three quarters reported “I have a little fear, with occasional thoughts but they don't really bother me” (44%) and “I am sometimes having fearful thoughts but I can usually manage these” (30%).

Exploring the longitudinal data, FCR severe rates remain relatively unchanged over the year with evidence of a slight improvement 8–6%. This serves to highlight how imbeded this fear can be for patients. Patients still wish to talk about FCR involving one in three at the start of the trial and one in five at the last consultation. The PCI prompt is potentially a useful adjunct to the conversation with the health professional as even with the passage of time it helps the patients to broach what can be a sensitive and distressing issue and this provides an opportunity to reassure on repeated occasions as prompted by the patient themselves.

There are several potential limitations of this work. Firstly, in the trial of those approached to be recruited, around one in five declined to participate. It might be that patients with an anxiety disorder are less likely to take part and that these are more likely to experience high FCR. Thus, this current analysis might underestimate the rates of FCR. The findings should have a reasonable level of generalisability, as although involving only two centers, 15 different consultants were involved, and the sample comprised 80% of eligible patients. The second limitation relates to the single FCR item question used as part of the HRQOL survey. It is recognized that this question will not be as reliable as other more comprehensive FCR measures (Thewes et al., 2012). However, this together with the PCI item on FCR gives a unique opportunity to analyse characteristics that might be predictive of higher FCR dysfunction. Because of the variety of different criteria and definitions of FCR, direct comparisons to this research with other studies should be treated with caution (Mirosevic et al., 2019). This should also be considered in any future work.

The potential value of using a prompt list (PCI) as part of the consultation is that patients might be more amenable to raise the FCR issue through this prompt, and also to discuss their fear in preference to discussing other psychological issues such as depression or anxiety. Fear of Cancer Recurrence is perceived as an understandable concern that both the patient and the clinician can relate to. When talking through natural and health concerns the clinician can gauge the extent of the fear and can offer additional support. A specific FCR intervention could be considered for those most distressed. Considerable work is now being conducted to develop interventions for those with high levels of FCR (Tauber et al., 2019). Further research is needed to carefully construct and evaluate the cost effectiveness of a tiered programme of support. However, a straightforward means to highlight the issue of FCR in routine oncology clinics, either by clinical characteristics, questionnaire caseness, or a prompt from the patient themselves, or a combination of these, could be a suitable starting point for both informal and formal Interventions, with the desired outcome being less distress and improved HRQOL.

## Data Availability Statement

The raw data can be made available by the authors, without undue reservation, to any qualified researcher through due process.

## Ethics Statement

The studies involving human participants were reviewed and approved by Liverpool. The patients/participants provided their written informed consent to participate in this study.

## Author Contributions

SR, DL, GH, and AK: study design and critical revision of the manuscript. SR, DL, and AK: data collection. All authors analysis, interpretation, drafting of the manuscript, and approved the final version for publication.

## Conflict of Interest

DL was employed by company Astraglobe Ltd. The remaining authors declare that the research was conducted in the absence of any commercial or financial relationships that could be construed as a potential conflict of interest.
