# Supplementary material for: Which Head and Neck Cancer Patients Are Most at Risk of High Levels of Fear of Cancer Recurrence
Source: Front Psychol. 2021 Jul 16;12:671366. doi: 10.3389/fpsyg.2021.671366 (PMC8322117; doi:10.3389/fpsyg.2021.671366)
Supplement: Supplementary file 1 [file Data_Sheet_1.PDF]

PCI

Aintree University Hospital NHS
NHS Foundation Trust
Where quality matters

Patient Concerns Inventory [PCI]
Head and Neck

Please choose from the list of issues you would specifically like to talk about in your consultation in clinic today. You can choose more than one option (tick boxes).

Physical and functional well-being:

- [ ] Activity
- [ ] Appetite
- [ ] Bowel habit
- [ ] Breathing
- [ ] Chewing/eating
- [ ] Coughing
- [ ] Dental health/teeth
- [ ] Dry mouth
- [ ] Energy levels
- [ ] Fatigue/tiredness
- [ ] Hearing
- [ ] Indigestion
- [ ] Mobility
- [ ] Mouth opening
- [ ] Mucus
- [ ] Nausea
- [ ] Pain in the head and neck
- [ ] Pain elsewhere
- [ ] Regurgitation
- [ ] Salivation
- [ ] Shoulder
- [ ] Sleeping
- [ ] Smell
- [ ] Sore mouth
- [ ] Swallowing
- [ ] Swelling
- [ ] Taste
- [ ] Vomiting/sickness
- [ ] Weight

Treatment related:

- [ ] Cancer treatment
- [ ] Regret about treatment
- [ ] PEG tube
- [ ] Wound healing

Social care and social well-being:

- [ ] Carer
- [ ] Dependents/children
- [ ] Financial benefits
- [ ] Home care/District nurse
- [ ] Lifestyle issues (smoking/ alcohol)
- [ ] Recreation
- [ ] Relationships
- [ ] Speech/voice/being understood
- [ ] Support for my family

Psychological, emotional and spiritual well-being:

- [ ] Appearance
- [ ] Angry
- [ ] Anxiety
- [ ] Coping
- [ ] Depression
- [ ] Fear of the cancer coming back
- [ ] Fear of adverse events
- [ ] Intimacy
- [ ] Memory
- [ ] Mood
- [ ] Self-esteem
- [ ] Sexuality
- [ ] Spiritual/religious aspects
- [ ] Personality and temperament

Others (please state):

....

....

....

....

NHS number:

Date:

EPRC

Edge Hill University

The following page gives you the opportunity to highlight people you may wish to talk to. Are there any people you would specifically like to talk with either in clinic or by referral?

Physical and functional well-being:

- ☐ Dental hygienist
- ☐ Dentist
- ☐ Oral rehabilitation team
- ☐ Dietician
- ☐ Physiotherapist
- ☐ Speech (swallow) and language therapist
- ☐ Occupational therapist
- ☐ Nursing staff
- ☐ Audiologist

Treatment related:

- ☐ Surgeon
- ☐ Oncologist/Radiotherapist
- ☐ Clinical Nurse Specialist

Social care and social well-being:

- ☐ Social worker
- ☐ General practitioner
- ☐ Financial advisor

Psychological, emotional and spiritual well-being:

- ☐ Chaplain
- ☐ Clinical psychologist
- ☐ Emotional support therapist

Others (please state):

Thank you for your time. All information is confidential. We found PCI has helped patients express issues in their clinic.

Version April 2017

© Edge Hill University and Aintree University Hospital retain the Intellectual Property Rights for the Patient Concerns Inventory.

NHS number:

Date:

PCI
